# The Efficacy of Filters

**Published:** 1889-07

**Authors:** 


THE EFFICACY OF FILTERS AND OTHER MEANS EMPLOYED TO PURIFY DRINKING WATER. A Bacteriological Study. By Charles G. Currier, M. D. Reprinted from *The Medical News*, April, 1889.

This pamphlet is of such great interest to all physicians that we are induced to give a considerable résumé of its contents. The author made practical and exhaustive tests under the microscope of all the different materials used in filters with a view of discovering how far they acted in arresting the passage of harmful bacteria. His labors may be summed up thus:

When chemicals are used to destroy bacteria in water, they must be used in such quantities that the water cannot be considered wholesome for drinking.

Boiling is a sure disinfectant, strongly insisted upon by the author.

Freezing does not destroy the bacteria to any great extent. Indeed, the addition of ice to drinking-water is apt to introduce new forms of bacteria contained in the ice.

Filters of charcoal, sand, etc., of small area, passing large quantities of water, are not efficacious in arresting bacteria. They only stop the visible sediment and render the water clear to the eye; hence their popularity. Yet they do not prevent the bacteria from penetrating. Unexpectedly they actually increase the number of bacteria in the water that is filtered. The bacteria simply lie in the interstices of such filters and propagate their species, which then appear in the water in increased numbers. This is true of all substances used as filters—charcoal, sand, sponge, prepared cotton, filtering paper, porous stone, porcelain, etc.

Asbestos board was remarkably successful in almost completely arresting the bacteria. Its value seemed to depend upon the fineness of the surface of the board.

Stone and porcelain filters were successful for the first few days in arresting the bacteria; but they, too, became charged, and then the bacteria appeared in the filtered water in largely increased numbers. On the whole, no effective purification of water can be expected from filters on the small scale.

Though sand will not prevent bacteria from passing, yet if it be expressly covered with a layer of silt, organic and inorganic material arrested by sand from a large body of flowing water, a very successful filter can be made that will effectually eliminate micro-organisms. Such filtering beds have been successfully worked in purifying the water of large cities. The filtering beds of Berlin are a conspicuous example. They are constructed in the following manner:

"Above the base of the filtering tanks is a layer of a foot of stones, gradually becoming smaller in size toward the upper surface, upon which is coarse gravel to the height of a foot or more; then upon this a little more than two feet of sand, which at the top is as fine as can be procured. When the filter-bed has been freshly cleaned, as is found necessary for it after being constantly used for a week or so, purified water is slowly backed into the filtering mass from below until this water, carrying up all the air with it, has reached the top of the upper layer. Then the ordinary river (or lake) water is made to flow very gently in to the depth of a metre. This is then allowed to stand for twenty-four hours. The nitrogenous or other particles, confervoid vegetation, and whatever else the water contains as sediment have then settled upon the upper portion of the fine sand without sinking deeper, and a delicate film is formed, which, with careful inlet and gentle pressure (never to exceed two metres of water), retains nearly all the bacteria of the water supplied, and prevents their passage, provided that the flow through is very regular and slow (never more than three metres in a day). Nearly all this separation of the bacteria is produced by the sedimental matter retained on the surface of the sand, so that, when the filter slows from clogging, it is found that less than half an inch of the upper layer of sand need be removed."

Prof. Leeds is the originator of another plan of filtration on the large scale. He adds a small portion of alum to the water to be filtered. The alum causes the suspended particles of silt, together with the organic matter, to form a deposit upon very fine sand through which the water is forced. The alum is present in the proportion of one part to one hundred thousand parts of water (1 : 100,000), and could not be detected by ordinary chemical tests. The author concludes:

"When filtering is really necessary, it is in general best for the community that it be done carefully on the large scale through sand-beds, upon which a fine layer of organic and inorganic matter is expressly produced by sedimentation, because of its valuable action in holding back the great majority of bacteria.

"A bad water filtered is less desirable than a pure water in its natural state. When, therefore, filtration is employed because of real danger of infection, the filtered water should, as a rule, be furthermore boiled, as the entire absence of sediment and cloudiness does not insure that the bacteria of disease may not have made their way through the filter."

In this pamphlet the author has, we think, given a clear and desirable presentation of the present status of the filter question. W. M. J.

